# Potential advantages of cell administration on the inflammatory response compared to standard ACE inhibitor treatment in experimental myocardial infarction

**DOI:** 10.1186/1479-5876-6-30

**Published:** 2008-06-12

**Authors:** Michele M Ciulla, Elisa Montelatici, Stefano Ferrero, Paola Braidotti, Roberta Paliotti, Giuseppe Annoni, Elisa De Camilli, Giuseppe Busca, Luisa Chiappa, Paolo Rebulla, Fabio Magrini, Lorenza Lazzari

**Affiliations:** 1 Istituto di Medicina Cardiovascolare, Centro di Fisiologia Clinica e Ipertensione, University of Milan, Italy; 2 Fondazione Ospedale Maggiore Policlinico, Mangiagalli e Regina Elena, IRCCS, Milan, Italy; 3 Cell Factory "Franco Calori", Milan, Italy; 4 II Cattedra di Anatomia Patologica, University of Milan, DMCO A.O. San Paolo, Milan, Italy

## Abstract

**Background:**

Bone Marrow (BM) progenitor cells can target the site of myocardial injury, contributing to tissue repair by neovascolarization and/or by a possible direct paracrine effect on the inflammatory cascade. Angiotensin Converting Enzyme inhibitors (ACE-I) are effective in reducing mortality and preventing left ventricular (LV) function deterioration after myocardial infarction.

**Methods:**

We investigated the short term effects of BM mononuclear cells (BMMNCs) therapy on the pro-inflammatory cytokines (pro-CKs) and on LV remodelling and compared these effects over a standard ACE-I therapy in a rat model of myocardial cryodamage.

Forty two adult inbread Fisher-F344 rats were randomized into three groups: untreated (UT; n = 12), pharmacological therapy (ACE-I; n = 14, receiving quinapril), and cellular therapy (BMMNCs; n = 16, receiving BMMNCs infusion). Rats underwent to a standard echocardiogram in the acute setting and 14 days after the damage, before the sacrifice. Pro-CKs analysis (interleukin (IL)1β, IL-6, tumor necrosis factor (TNF)α was performed (multiplex proteome arrays) on blood samples obtained by direct aorta puncture before the sacrifice; a control group of 6 rats was considered as reference.

**Results:**

Concerning the extension of the infarcted area as well as the LV dimensions, no differences were observed among the animal groups; treated rats had lower left atrial diameters and higher indexes of LV function. Pro-Cks were increased in infarcted-UT rats if compared with controls, and significantly reduced by BMMNCs and ACE-I ; TNFα inversely correlated with LV fractional shortening.

**Conclusion:**

After myocardial infarction, both BMMNCs and ACE-I reduce the pattern of pro-Ck response, probably contributing to prevent the deterioration of LV function observed in UT rats.

## Background

After myocardial infarction the host response includes inflammatory response and cytokine production, that modulate tissue repair and response and are determinant for the patient outcome [1].

Experimental animal studies have provided evidence that bone marrow (BM) progenitor cells are capable to selectively target the site of myocardial injury [2] and contribute to tissue repair [3]. More recently the interest has focused on the hypothesis that BM progenitors could ameliorate left ventricular (LV) remodeling following myocardial infarction by continuing to differentiate along the hematopoietic lineage [4]. But currently no evidences have been provided demonstrating that in animals transplanted with different stem or progenitor cell populations the damaged area has been partially or completely regenerated by new cardiomyocytes. Unfortunately the homing have been shown to be transient [5] and only few transplanted cells have been found in the site of the myocardial injury [6] even if cardiac functions have been observed to ameliorate. Therefore, other possible explanations have been proposed in order to clarify the mechanisms underlying the positive results observed in animals models and humans. In this context, a possible mechanism of the BM cell therapy benefit could derive either by new vessels formation [7,8] at the infarct site and/or by a direct paracrine effect on the inflammatory cascade [9].

On the other hand, several clinical studies based on cell therapy with stem and progenitors cells are producing interesting but still debated results [10-12].

Angiotensin Converting Enzyme inhibitors (ACE-I) are considered a first line therapy following myocardial infarction in humans because of their demonstrated efficacy in reducing mortality and preventing deterioration of LV function [13], partially due to a reduction in cardiac cytokine expression in the subacute and chronic period after the injury [14,15].

In this general context no studies are available comparing the efficacy of BM progenitors cells with conventional ACE-I therapy after myocardial infarction.

In the hypothesis that the efficacy of BM mononuclear cells (BMMNCs) after myocardial infarction is mediated by a paracrine mechanism, in this study we investigated the short term effects of BMMNC therapy on the pro-inflammatory cytokine (pro-Ck) signaling pathways and on LV remodelling markers and compared these effects over a standard ACE-I pharmacological therapy in a rat model of myocardial cryodamage. By using an animal model that allows to mimic the autologous infusion of BM progenitors avoiding immunosuppression and an experimental myocardial injury procedure that facilitates the association of transplanted cells with the infarcted versus the non infarcted areas [16], we have shown, for the first time, that peripherally injected BMMNCs significantly reduce the pro-Ck response.

## Methods

### Animal model and experimental myocardial cryoinjury

**Figure 1:**
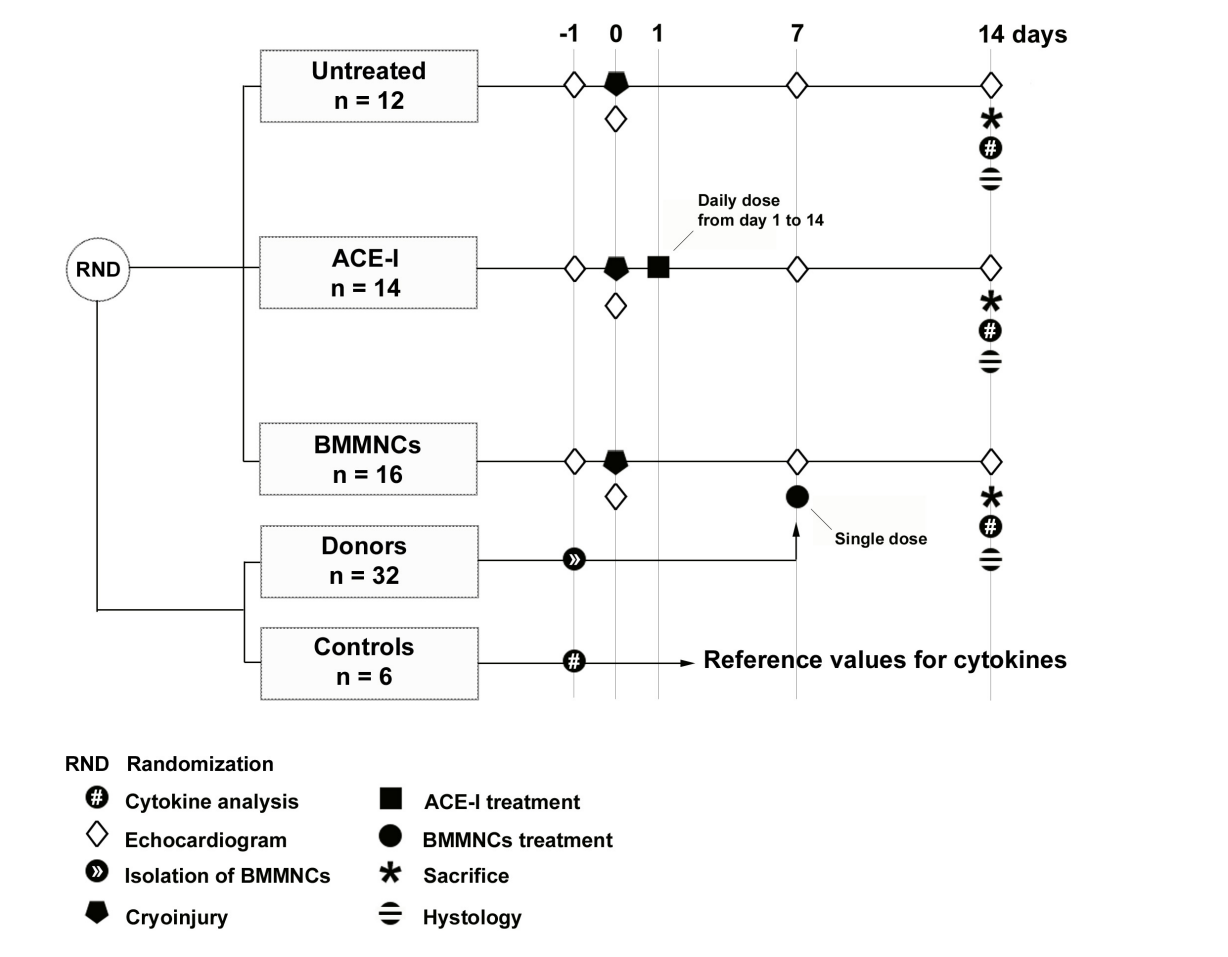
Design of the experiment.

A total number of 42 male adult inbred rats (Fisher-F344; Charles River Laboratories, Italy) weighting 200–250 g. were studied. Animals were housed and handled in accordance with the "Guide for the Care and Use of Laboratory Animals" [17]. To ensure the permanent identification, at the arrival each rat was implanted with a microchip device (MUSICC, AVID Microchip, Barcelona, Spain). Experimental myocardial cryoinjury was produced by freeze-thaw technique, previously described in detail [16], that allows producing a predictable cardiac lesion. The animals were therefore randomized into three groups: untreated group receiving no treatment (UT; n = 12), pharmacological therapy group treated with quinapril (ACE-I; n = 14), and cellular therapy group treated with BMMNCs infusion (BMMNCs; n = 16) (Figure 1).

### Isolation, characterization, labeling and administration of BMMNCs

Thirty two rats were used as donors of BMMNCs; isolation of cells from the femoral and tibial bones of donor rats and labeling and administration in recipient rats (n = 16) were performed according to a previously described method [2]. The number of BMMNCs expressing the CD34 surface marker and the viability of pooled cells were assessed by flow-cytometry analysis of 100,000 events per sample (FACScan, Becton Dickinson, Franklin Lakes, NJ). Before administration, cells were labeled with a red fluorescent cell linker (PKH26 dye, Sigma, St. Louis, MO). Seven days after injury, the recipient rats were prepared for the infusion of a single dose of BMMNCs. Rats were anesthetized (ketamine, 2 g/Kg body weight i.p.) and the femoral vein was surgically isolated and exposed. The BMMNCs suspension (500 μL) containing 20 ± 2.1 × 10^6 ^cells, with a mean of 1.03 ± 0.2% CD34+, was infused using a sterile tuberculin syringe with a 23G needle. The wound was then closed with 5-0 vicryl sutures.

### ACE-I treatment

The ACE-I quinapril, with a known high heart tissue ACE affinity [18], was administered orally in drinking water and adjusted to reach the dose of 1 mg/kg/die. The treatment was started the day after myocardial cryoinjury and followed for 14 days, with an overall administation of 1.75 mg of quinapril in each treated rat.

### Echocardiographic examination

In vivo heart dimensions, function and infarct size were evaluated by standard echocardiography by using an echocardiographic system (GE Vivid 7, Wisconsin, USA) equipped with a 13 MHz linear array probe (focus depth set at 1.0 cm). Rats were examined under diethyl ether anesthesia, in the left lateral decubitous position, before the cryodamage, in the acute setting (within 24 hours after the injury) and before the sacrifice, 14 days after the injury. Infarct size was assessed at the end of the study by planimetry on 2D, short-axis acquired in real time images of the LV according to a previous described procedure [2].

### Pathological examination

Fourteen days after the myocardial damage rats were sacrificed with an overdose of sodium pentobarbital. The heart was exposed through a sternotomy. The heart was then arrested in diastole with an injection of potassium chloride (10 mEq) and the site of myocardial injury was identified by gross examination. In order to obtain adequate material for the different investigations, the heart was sampled, the infarcted region was transversely cut into two sections of about 5 mm in thickness and frozen in cryostat embedding medium (Bio Optica, Milan, Italy). From each frozen block 5 μm thick cryostatic sections were obtained and used to evaluate the morphology of the infarcted region and to perform the immunoreactions. Immunostaining was performed by using the following primary antibodies at the following dilutions: Sarcomeric Actin, 1:200 from Dako (Dako A/S, Glostrup, Denmark); CD34, 1:100 (clone ICO115) purchased from Santa Cruz Biotechnology Inc. (Becton Dickinson, Santa Cruz, CA, USA).

### Serum cytokine assay

Cytokine profile analysis was performed by chemiluminescent enzyme immunometric assay (SearchLight Proteome Array, USA) on 50 ml serum samples (diluted 1:5) obtained by direct puncture of the aorta 14 days after the myocardial damage, before the sacrifice. The array layout included the following 9 cytokines: interleukin (IL)-1α, IL-1β, IL-2, IL-4, IL-6, IL-10, interferon (IFN)γ, tumor necrosis factor (TNF)α, granulocyte-macrophage colony-stimulating factor (GMCSF); of them, the pro-CKs included in the study were IL-1β. IL-6, TNFα. The lower detection limit for each cytokine was set according to the manufacturer. Normal cytokine values were obatined from an age and sex matched control group (n = 6).

### Morphometrical analyses

The homing of the injected population to the tissue target and their phenotype was confirmed by immunofluorescence and immunohistochemistry using a previously described approach [8]. Briefly, immunofluorescence was revealed with a multi-photon confocal microscope (Bio-Rad Radiance 2100, Carl Zeiss, Germany) using HeNe laser (543 nm). In each section at least 6 fields (20×) corresponding to the infarcted/non-infarcted areas were selected, acquired and stored on a personal computer (Power Mac G4, 867 Mhz, 640 MB RAM, Apple, Cupertino, USA) in JPEG format (5:1) by using a CCD camera (COHU 2200, Cohu Inc., San Diego, CA, USA) coupled to an analog-to-digital acquisition board (Dazzle DV-Bridge, SCM Microsystems, Munich, Germany) operating at 32 bits/pixel on a 764 × 560 pixel matrix. Finally, stored images were analyzed to confirm the homing of PKH26+/CD34+ cells by using a computer integrated image-processing software according to a previously described procedure [8].

### Immunohistochemistry

Sections from formalin fixed, paraffin embedded rat IMA myocardial samples were incubated with Goat anti Rat TNFα polyclonal antibody diluted 1:200 (Santa Cruz Biotechnology Inc. Becton Dickinson, Santa Cruz, CA, USA). Previous antigen retrieval was obtained by 30 min in 90°C citrate buffer. Positive (lymph node) and negative (healthy rat myocardium) controls were added.

### Statistical analysis

Data were analyzed using a computer statistical software (SPSS – Rel 11; SPSS Inc., Chicago, Illinois, USA). Infarcted areas are reported as mean ± standard deviation. The effects of the two treatments on LV function and pro-Cks were tested with ANOVA with Fisher's post hoc test. The serum levels of each cytokine were correlated with LV function parameters by linear regression analysis. All statistics were considered significant with p value less than 0.05.

## Results

At the end of the study, the mean area of infarction in the studied animals measured in vivo by echocardiography was 21.7 ± 3.2% of the transverse LV free wall. Gross examination of the excised hearts confirmed the presence of a regular non-transmural scar of the entire LV free wall. Histology confirmed the presence of a hemorrhagic infarct, without transition zones from non-infarcted to infarcted areas. No significant differences were found when comparing the infarcted area in BMMNCs versus ACE-I treated rats (23.4 ± 3.2 vs 20.4 ± 2.7%, respectively; p = 0.08). Confocal microscopy confirmed that in BMMNCs treated rats PKH26+/CD34+ elements targeted the infarcted areas (mean 13.9 ± 4.6 cells per field).

**Figure 2:**
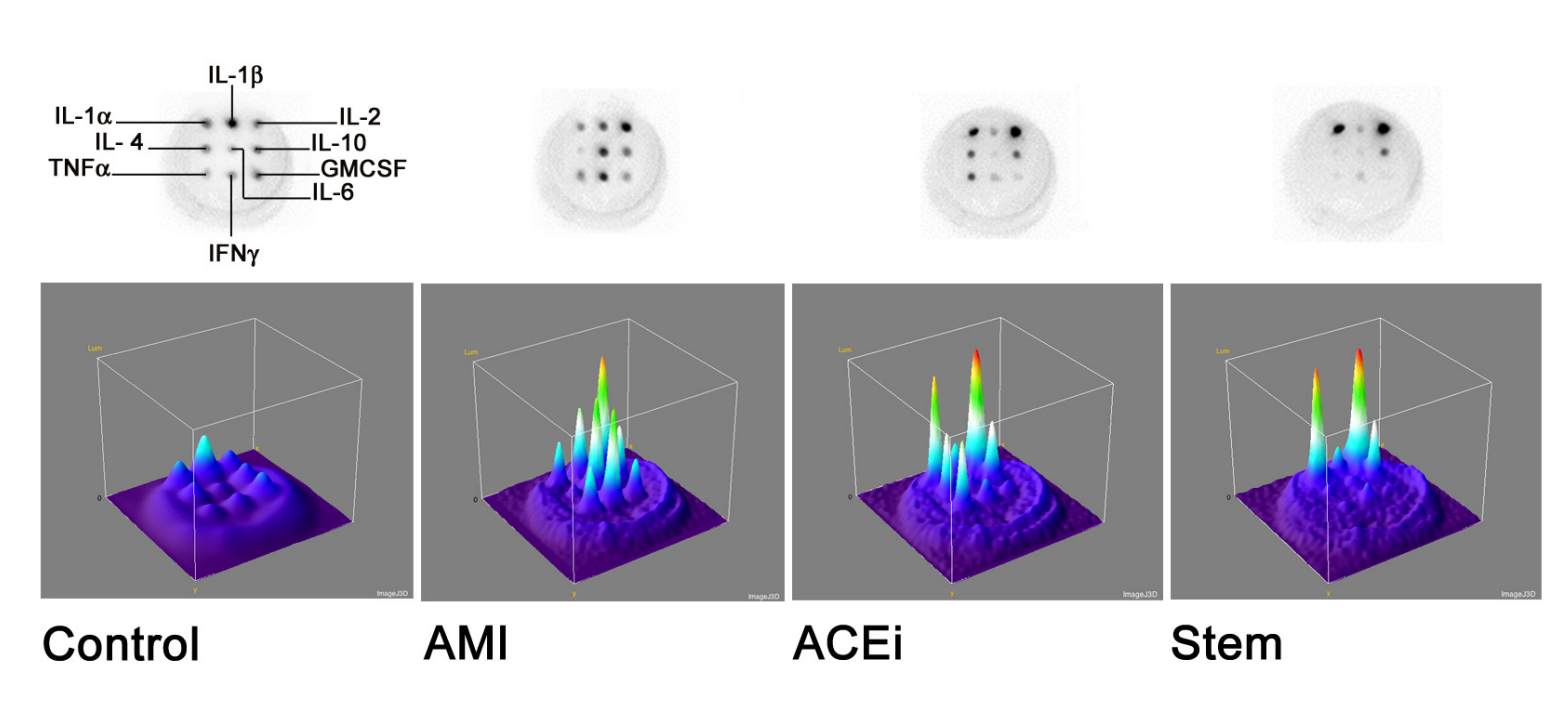
*Top panels*. Representative original chemiluminescent images obtained from the studied rats. The loading scheme of the cytokines is shown on the first top left image. *Bottom panels*. Surface plots obtained from the corresponding chemiluminescent images showing on the z axis the relative intensity of the responce; the luminance of the image is interpreted as height of the plot. In the AMI plot (untreated rat) is clearly evident the pro-Cks increase if compared with the ACE-I and Stem plot where, on the contrary, the anti-inflammatory Cks are prevalent. Plots are obtained by using Interactive 3D surface plot v2.1 by K. Barthel a specific software developed for ImageJ v1.37, a freeware image analysis package developed by W. Rasband. AMI: acute myocardial infarction.

Fourteen days after the experimental myocardial damage a significant increase in the serum levels of pro-CKs was found in infarcted-UT rats if compared with non-infarcted control rats (Table 1). Treatment with ACE-I and BMMNCs resulted in significant lower serum levels of pro-CKs in comparison with infarcted-UT rats (Table 2). The extent of these differences in the pro-CKs were similar in both treatment groups for IL-1β, while for IL-6 and TNFα it was greater in BMMNCs treatment, even if the difference between the two treatment was not statistical significant. When examining the other cytokines included in the array, an effect was observed only on IL-4 that was significantly reduced in both treatment groups if compared with untreated rats, with no statistically significant differences between BMMNCs and ACE-I (IL-4: Controls 1.3 ± 0.8 pg/ml; UT 31.1 ± 9.1 pg/ml; ACE-I 9.6 ± 23.6 pg/ml; BMMNCs 1.5 ± 2.6 pg/ml; UT vs Controls, p = 0.003; UT vs ACE-I, p = 0.005; UT vs BMMNCs p = 0.0001; ACE-I vs BMMNCs, p = ns). The patterns of pro-Cks responses are shown in figure 2.

**Table 1:** Pro-inflammatory cytokines in untreated rats: comparison with controls

	Controls	Untreated	ANOVA
	n = 6	n = 12	p value
IL-1β pg/ml	6.51 ± 6.46	164.20 ± 147.70	0.0020
IL-6 pg/ml	1.76 ± 1.28	954.22 ± 567.23	0.0002
TNFα pg/ml	1.96 ± 1.73	596.45 ± 376.81	0.0001

**Table 2:** Pro-inflammatory cytokines in treated rats: comparison with untreated.

	Untreated	ACE-I	ANOVA^#^	BMMNCs	ANOVA^§^
	n = 12	n = 14	p value	n = 16	p value
IL-1β pg/ml	164.20 ± 147.70	42.66 ± 84.49	0.0026	42.06 ± 65.75	0.0026
IL-6 pg/ml	954.22 ± 567.23	219.65 ± 487.04	0.0021	110.43 ± 309.98	0.0002
TNFα pg/ml	596.45 ± 376.81	143.64 ± 264.36	0.0012	66.23 ± 127.89	<0.0001

^# ^Untreated vs ACE-I

^§ ^Untreated vs BMMNCs

LV dimensions in both treated groups were not substantially different if compared with untreated, while left atrial diameters were lower in treated rats. Diastolic and systolic indexes of LV function were still within the normal range in all groups, even if fractional shortening (FS) was statistically significant higher in both treated rats; finally, ejection fraction (EF) was higher in ACE-I treated rats (Table 3). When comparing serum pro-Ck levels in infarcted animals with LV function, an inverse significant correlation was found between TNFα and FS (r = 0.84; p = 0.008).

**Table 3:** Effects of treatment on LV dimensions and function

**-1 days**				**0 days #**			**7 days**			**14 days**		
	**UT**	**ACE-I**	**BMMNCs**	**UT**	**ACE-I**	**BMMNCs**	**UT**	**ACE-I**	**BMMNCs**	**UT**	**ACE-I**	**BMMNCs**

**LAd mm**	2.3 ± 0.2	2.2 ± 0.2	2.2 ± 0.3	3.2 ± 0.3	2.7 ± 0.4*	3.1 ± 0.4	3.4 ± 0.2	2.7 ± 0.4**	3.1 ± 0.3	3.5 ± 0.3	2.7 ± 0.4*	2.6 ± 0.6*
**LVEDd mm**	5.0 ± 0.5	5.1 ± 0.1	5.1 ± 0.2	5.5 ± 0.5	5.2 ± 0.2	5.4 ± 0.3	5.5 ± 0.7	5.3 ± 0.2	5.4 ± 0.2	5.6 ± 0.7	5.4 ± 0.2	5.3 ± 0.3
**E/A**	3.9 ± 0.1	3.9 ± 0.1	3.8 ± 0.1	3.7 ± 0.5	3.6 ± 0.4	3.3 ± 0.3	3.5 ± 0.4	3.6 ± 0.5	3.0 ± 0.3	3.4 ± 0.5	3.4 ± 0.6	2.6 ± 0.4
**FS %**	50.1 ± 1.0	50.2 ± 2.5	49.9 ± 1.7	39.9 ± 4.8	47.4 ± 3.5**	42.2 ± 3.8	38.0 ± 3.6	46.8 ± 3.6**	39.6 ± 3.6§	37.4 ± 3.4	45.3 ± 3.6**	43.5 ± 3.9*
**EF %**	80.0 ± 0.8	81.9 ± 3.4	81.8 ± 1.3	71.8 ± 5.5	80.1 ± 3.1**	76.4 ± 4.6	71.7 ± 5.2	81.3 ± 3.6**	77.8 ± 4.4	72.2 ± 4.8	81.1 ± 3.6**	77.8 ± 4.1

LAd = Left Atrial Diameter; LVEDd = Left Ventricular End Diastolic Diameter; E/A = ratio between the velocities of E and A waves of the mitral diastolic flow; FS = Fractional Shortening.

# data on day 0 refer to echocardiograms performed 6–24 hours from injury.

*p = 0.05 vs UT ; **p = 0.01 vs UT; §p = 0.01 vs ACE-I

**Figure 3:**
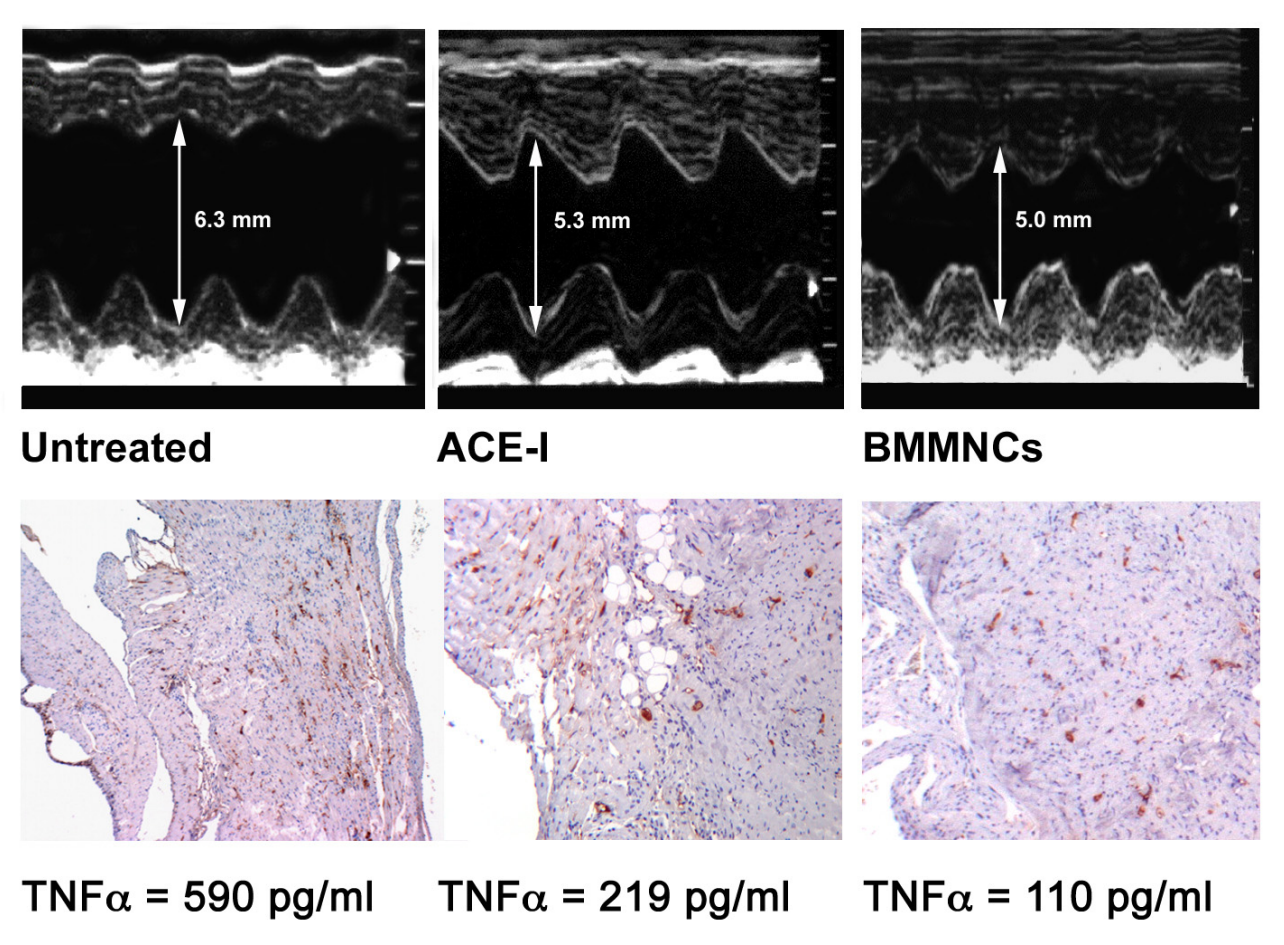
*Top panels*. Original M-mode echocardiographic tracings showing left ventricular end diastolic diameters (LVEDd), respectively in an Untreated (left), an ACE-I (centre) and a BMMNCs (right) treated rat. *Bottom panels*. In the same rats, immunohistochemistry images showing TNFα positive elements (DAB+) and the corresponding TNFα.serum levels.

The effects of treatments on TNFα circulating levels were also confirmed locally by the immunohistochemistry (Figure 3).

## Discussion

Following the hypothesis that the mechanisms of potential effects of BM progenitors after myocardial infarction can be due also to a direct paracrine effect on the inflammatory cascade, in this study for the first time we have studied the secretion of pro-Cks in the subacute phase of the damage (14 days from the injury) after peripherally injected BMMNCs and we compared these results with the ones obtained with the conventional pharmacological treatments.

It is well known that cytokines such as TNF-α or IL-1β and IL-6 are not constitutively expressed in the normal heart; they are secreted after myocardial ischemic injury, with very important acute and chronic effects on myocardium, including myocyte apoptosis and hypertrophy, defects on contractility and inflammatory signal transduction, activation of matrix metalloproteinases and collagen formation, integrin regulation, angiogenesis and progenitor cells mobilization.

In this context, we have shown that peripherally injected BMMNCs reduce the pro-Cks response in the subacute phase of the damage (14 days from the injury), when it is documented to be significantly increased [19]. And in particular, the administration of BMMNCs decreased the serum levels of IL-1β, IL-6 and TNFα that are from fourfold to ninefold lower that in untreated rats.

Moreover, in these experiments, our model of cryoinjury has been shown to be reliable in terms of inflammatory response since the levels of these inflammatory cytokines are very low in controls with a prompt increase after the injury (7 days) of about 25 to 540 fold.

The role played by ACE-I in the modulation of inflammatory response is still debated. While previous *in vivo *studies using ACE-I with low [20] or mild [15] tissue potency, according to Dzau et al. [21], didn't find a significant anti-cytokine effect, in agreement with other authors [14] we observed a decrease in cytokine serum levels also in the ACE-I treated group, indicating that quinapril is able to reduce the pro-Cks release when introduced early (same day of the injury). This finding supports the evidence that some of the effects observed with quinapril are related to its high affinity for tissue ACE.

When comparing the effects of a cell therapy approach (BMMNCs) versus the pharmacological one (ACE-I), we observed that the reduction in the pro-Cks was similar in both treatment groups for IL-1β, while for IL-6 and TNFα it was greater in BMMNC treatment, even if the difference between the two treatment was not statistical significant.

Furthermore, since in our model the progression to heart failure is known to be a slow process [16], we didn't find a significant impairment of LV function in any of the infarcted rats; nonetheless, a favorable effect was detected over FS that was higher in both treatment groups and over EF that was higher in ACE-I treated rats. This finding is coherent with the lower atrial dimensions in treated rats reflecting a better LV filling pattern, and it could depend at least in part on the modulation of the pro-Cks cascade, as shown by the inverse correlation between TNFα and FS. At this regard, in a recent paper by Boyle et al. [22] the use of a combined experimental approach including ACE-I, β-blocker and BM precursors, with known effects on the pro-Cks cascade following coronary artery ligation in rats, resulted in the prevention of deterioration of LV function from day 2 to day 16 post-myocardial infarction.

It should be noticed that our model of myocardial damage, previously described in detail [16,23], differs in some aspects from the temporary ligation of the artery [24] but has been proven to facilitate the association of the injected cells with the infarcted versus non-infarcted areas and to resemble a typical anterior non transmural myocardial infarction [25], including the inflammatory response [19].

## Conclusion

In the perspective of a future application, the potential benefit of the use of cell therapy after myocardial infarction seems to be, at least in part, related to the modulation of the inflammatory response consisting in lowering the serum pro-Cks, that are known to contribute to myocardial apoptosis, necrosis, and scar formation [26]. These actions are associated with a concurrent effect on the antiinflammatory cytokines, namely the regulation of IL-4 function, possibly via a direct effect on the lymphocyte T population. The effects of the cell therapy approach on the cytokine system associated with the previously demonstrated contribution to form CD34+ independent vascular structures [8,27] are probably the main mechanisms of benefit of cell therapy.

## Competing interests

The authors declare that they have no competing interests.

## Authors' contributions

MMC conceived the study, partecipating in its design, coordination and image analysis, EM carried out cell isolation, characterization, labelling and serum cytokine analysis, RP conducted the statistical analysis and drafted the manuscript, SF, PB, EDC carried out tissue sampling and hystopathological examinations, GA, GB and LC, conducted the animal experiments, GA and LC supervised pharmacological treatments, MMC and GA carried out echocardiograms, FM, LL contributed to the study design and critically revised the manuscript and. All authors read and approved the final manuscript.
